# Treatment of Systemic Lupus Erythematosus using BCMA-CD19 Compound CAR

**DOI:** 10.1007/s12015-021-10251-6

**Published:** 2021-08-30

**Authors:** Wenli Zhang, Jia Feng, Andrew Cinquina, Qingwen Wang, Haichan Xu, Qian Zhang, Lihua Sun, Qi Chen, Lei Xu, Kevin Pinz, Masayuki Wada, Xun Jiang, Yupo Ma, Hongyu Zhang

**Affiliations:** 1 Department of Hematology, Peking University Shenzhen Hospital, Shenzhen, People’s Republic of China; 2 Research & Development Division, iCell Gene Therapeutics LLC, Long Island High Technology Incubator, 25 Health Sciences Drive, Stony Brook, NY 11790 USA; 3 Department of Rheumatism and Immunology, Peking University Shenzhen Hospital, Shenzhen, People’s Republic of China

To the Editor:

Hematopoietic stem cell transplantation (HSCT) has been used to “reset” the immune system to protect against self-reactivity through the eradication of lymphoid cells and memory immune cells responsible for the longevity of self-reactivity. However, the conditioning regimen of high-dose chemotherapy or chemoradiotherapy used in HSCT is unlikely to completely deplete the population of memory immune cells and may not be practical given the severity of its toxicity. While autologous HSCT, which involves the use of the patient’s own stem cells, is safer because it avoids complications from graft-versus-host disease (GVHD), allogeneic HSCT, which involves the transfer of stem cells from a healthy donor, seems to provide better and longer-lasting outcomes. The GVHD associated with allogeneic HSCT is caused by differences in HLA between the graft and recipient, leading to an attack on recipient cells by donor cells. It appears that GVHD may play a role in the further destruction of the recipient’s immune cells responsible for autoimmunity, thereby reducing the risk of relapse. Earlier relapse seen with autologous HSCT likely may result from the survival of autoreactive residual memory immune cells [1]. Additionally, allogeneic HSCT has also been shown to induce remission in lupus-like autoimmune disease in animal models [2]. However, while allogeneic HSCT has greater success in “resetting” the immune system, allogeneic HSCT can have a mortality rate as high as 20%, can be associated with severe negative effects on the patient’s quality of life, and may not be the optimal method for an immune system reboot.

Instead, we have used a CAR-based approach to “reset” the antibody-producing lineages to treat autoimmunity through dual targeting of B cells and long-lived plasma cells (Fig. 1). Previously, we have shown that a CD19-BCMA compound CAR (abv. cCAR) is capable of profound reductions in donor-specific antibody levels through dual targeting of CD19 on B cells and BCMA (CD269) on plasma cells [3]. This dual action may be ideal for the treatment of autoimmunity, as previous clinical studies using anti-B-cell therapy alone with strategies such as the anti-CD20 antibody Rituximab have been disappointing. While CD19 CAR T cell therapy led to improved outcomes in mouse models of lupus, autoreactive antibodies were eliminated only when given at an early course of disease [4, 5]. Later in the disease course, long-lived plasma cells may accumulate and lead to persistent autoantibody production despite B-cell depletion [6, 7]. While early CD19-based therapy may prevent accumulation of these autoreactive plasma cells, administration of CD19 CAR T cells later in disease course would be ineffective at removing already formed plasma cells. As clinical therapy would likely begin later in disease course when there is a substantial autoreactive plasma population, dual targeting of B and plasma cells may have more success in “resetting” the antibody-producing population, leading to more complete removal of autoantibodies and improved clinical outcomes.

**Fig. 1:**
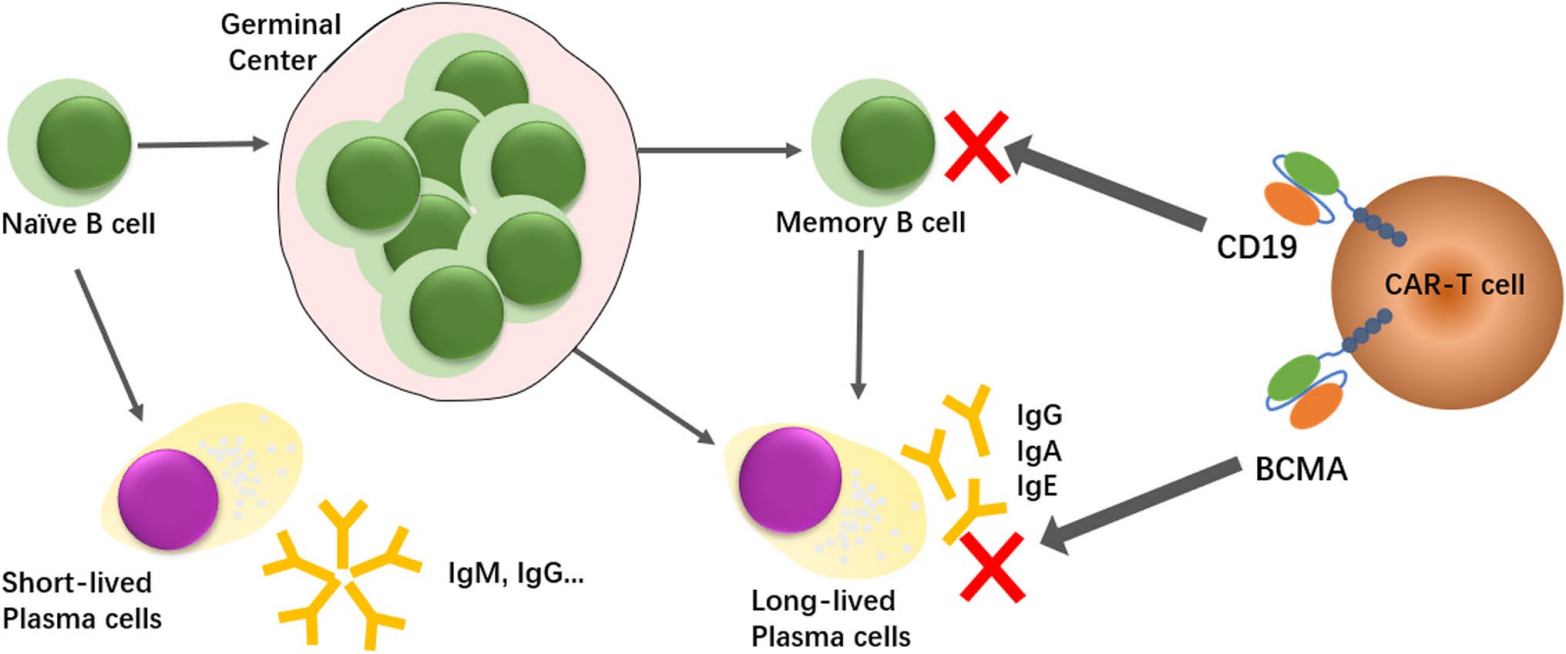
Mechanism of cCAR. The cCAR construct is a two-unit CAR composed of a complete BCMA-CAR fused to a complete CD19-CAR (also called CD19B-CAR) by a self-cleaving P2A peptide, enabling independent expression of both CAR receptors separately on the T cell surface. This allows cCAR to target two long-lived antibody-producing “root cells” – CD19 + memory B-cells and BCMA + plasma cells

We report the case of a 41-year-old female with 20-year-history of SLE who was recently diagnosed with stage IV diffuse large B-cell lymphoma (DLBCL). R-CHOP therapy was discontinued after severe side effects, and the patient was enrolled in the first clinical trial (NCT04162353) against SLE using cCAR. Prior to cCAR therapy, the patient received a pretreatment regimen of fludarabine and cyclophosphamide and discontinued prednisone therapy. Afterwards, 5.3 × 10^6^/kg cCAR T cells were infused, which expanded within two months of treatment (Fig. 2A). B-cells remained at undetectable levels until Day 198 post cCAR, with recovery to normal levels 9 months post-cCAR (Fig. 2B). Despite the discontinuation of prednisone, the patient’s C3 and C4 levels remained within normal limits (Fig. 2C), indicating the absence of complement activation. Various anti-nuclear antibody (ANA) titers were highly elevated prior to cCAR therapy; however, nuclear, cytoplasmic, and granular ANA reached undetectable levels by 9, 12, and 37 weeks post-cCAR, respectively (Fig. 2D). These reductions in ANA corresponded with marked reductions in total immunoglobulin levels (Fig. 2E, F). Immunoglobulin levels did not reach undetectable levels with cCAR, likely because the patient received prophylactic treatment with IVIG to limit chance of opportunistic infection during the period of B-cell aplasia. Despite the recovery of B-cells by Day 260 post-cCAR (Fig. 2B), ANA titers remained undetectable after 37 weeks (Fig. 2D), indicating continued absence of autoantibody production. In addition to removing pathogenic autoantibodies, the cCAR T cells also had a significant effect on the patient’s DLBCL. Prior to treatment, bone biopsies determined the presence of atypical lymphocytes and plasma cells in the bone marrow. PET-CT demonstrated the location of the tumors and indicated spread to the regional right external iliac lymph node (Supplementary Fig. 1A). Four months after cCAR infusion, repeat PET-CT demonstrated an absence of lesions (Supplementary Fig. 1B). Depletion of plasma cells in the marrow after cCAR therapy was also confirmed by bone marrow aspirate and subsequent flow cytometry (Supplementary Fig. 1C, 1D).

**Fig. 2:**
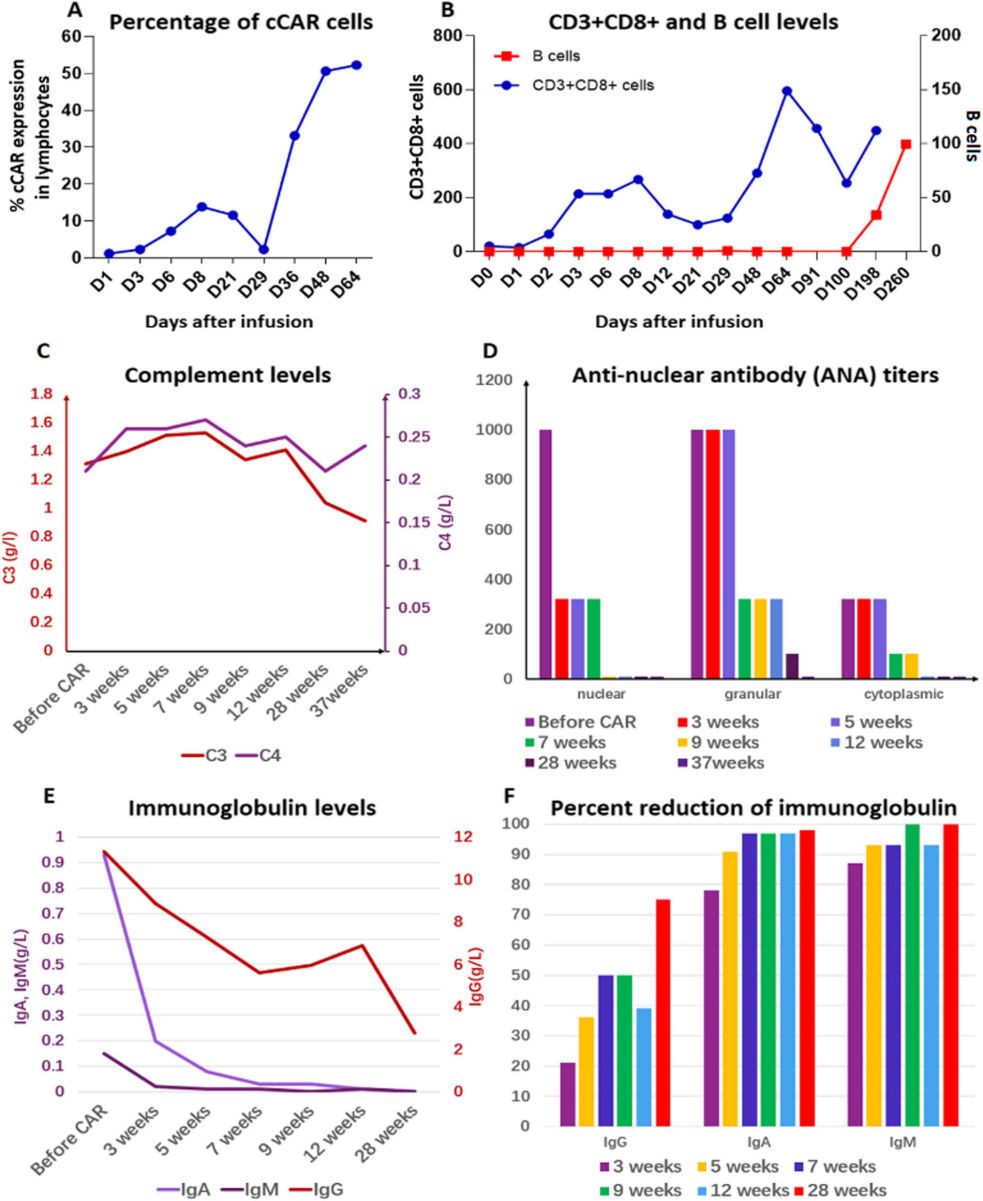
SLE remains inactive in patient after cCAR T cell injection (**A**) After the infusion of 5.3 × 10^6^/kg cCAR T cells, the first peak of cCAR T cells in the peripheral blood accrued at 7 days after cCAR infusion, and marked expansion of cCAR T cells was seen during the two months of treatment. Measured in percentage of total lymphocytes by flow cytometry analysis. (**B**) The number of B cells and CD3 + CD8 + cells were also monitored during the treatment. CD3 + CD8 + cells reached their first peak 7 days after cCAR treatment at 267 cells/μL and a second peak 2 months after treatment at 595 cells/μL. B cell numbers remained undetectable for three months post-cCAR and returned to normal levels 9 months after cCAR therapy. Measured in 10^6^ cells/L. (**C**) Complement levels of C3 and C4 were measured before and after cCAR treatment. Levels remained normal despite discontinuation of immunosuppressive therapy. Normal range for C3 and C4 are 0.8–1.6 g/L and 0.16–0.48 g/L, respectively. (**D**) Anti-nuclear antibody (ANA) titers were measured before and after cCAR treatment. Levels of nuclear ANAs were highly elevated prior to treatment, declined in the first few weeks, and remained undetectable after 12 weeks. Granular and cytoplasmic ANA had similar responses. (**E**) Immunoglobulin levels of IgG, IgM, and IgA were measured before and after cCAR therapy. All immunoglobulin levels decreased demonstrating a decrease in B cells and immunoglobulin-producing plasma cells. (**F**) Representation of the decline in immunoglobins from (**E**) as percent reduction from their levels before cCAR treatment

23 months post-cCAR, the patient’s SLE remains stable and DLBCL in remission despite receiving no additional immunosuppressive or chemo/radiotherapy. While previous mouse models of lupus demonstrated inability to completely remove autoantibodies unless treatment was begun early in disease, cCAR was able to reduce ANA titers to undetectable levels in this patient with a twenty-year history of SLE. The success of cCAR highlights the importance of targeting long-lived plasma cells which can continue producing autoantibodies despite B-cell depletion from CD19 CAR T-cells or Rituximab. Additionally, the recovery of B cell levels indicates that the aplasia is temporary, alleviating concern over potential prolonged immunodeficiency, and the continued absence of ANA titers demonstrates autoantibody production does not renew when B cell numbers return to normal. This suggests that cCAR can effectively “reset” the antibody-producing “root” B cells and plasma cells, leading to favorable outcomes in autoimmunity. This strategy offers a potentially safer and more targeted approach to “reset” the immune system than HSCT and could have profound implications for the treatment of SLE and other antibody-mediated disorders.

## Supplementary Information

Below is the link to the electronic supplementary material.

Supplementary file1 (DOCX 2199 KB)

Supplementary file2 (DOCX 768 KB)
