# Supplementary material for: Treatment of Systemic Lupus Erythematosus using BCMA-CD19 Compound CAR
Source: Stem Cell Rev Rep. 2021 Aug 30;17(6):2120–3. doi: 10.1007/s12015-021-10251-6 (PMC8599262; doi:10.1007/s12015-021-10251-6)
Supplement: Supplementary file 1 — Supplementary file1 (DOCX 2199 KB) [file 12015_2021_10251_MOESM1_ESM.docx]

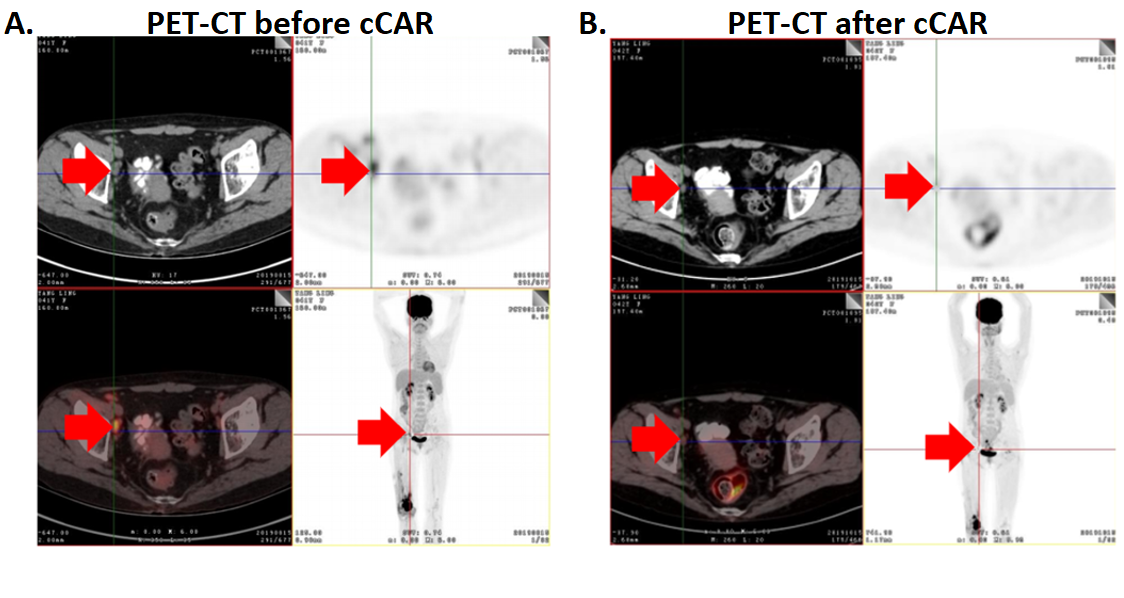

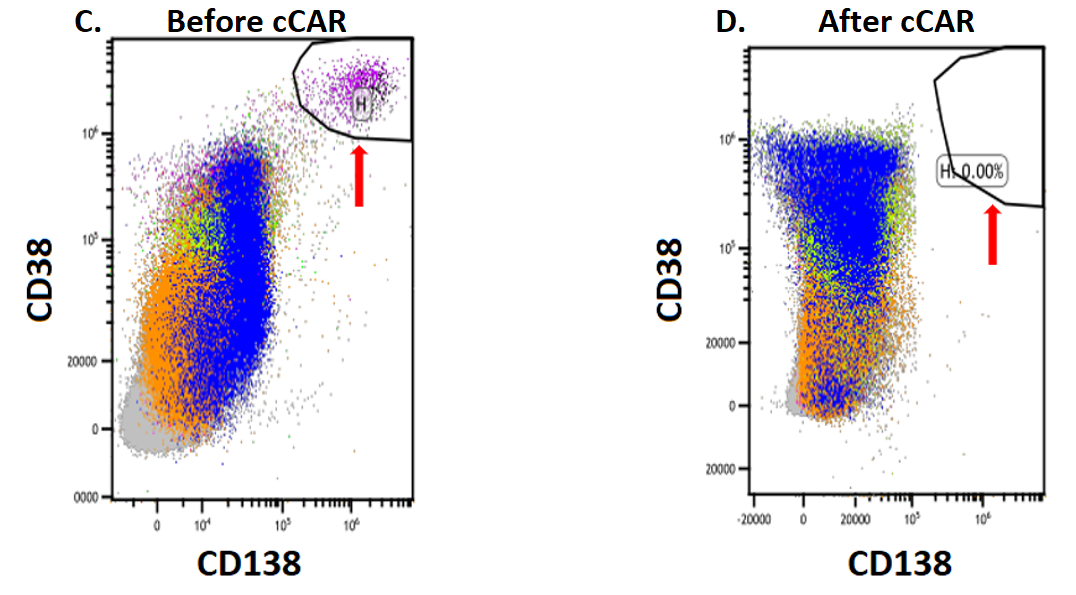


**Supplementary Figure 1**- **cCAR T cell infusion leads to complete remission of stage IV diffuse large B cell lymphoma (DLBCL)**. (**A.**) Patient was diagnosed with stage IV diffuse large B cell lymphoma (tumor in bone, inguinal lymph nodes, and bone marrow). Fluorodeoxyglucose uptake on positron-emission tomography computed tomography (PET-CT) demonstrate the sites of tumor. Red arrows point to right external iliac lymph node tumor. (**B.**) Patient received 5.3 x 10^6^/kg cCAR T cells and a repeat PET-CT was performed four months later. This showed an absence of lesions, indicating a complete remission. Red arrows point to same locations as in (**A.**). (**C.**) Bone marrow aspirate was performed and analyzed by flow cytometry. A population of plasma cells expressing CD38 and CD138 was detected before cCAR therapy. (**D.**) 5 weeks after cCAR therapy, a repeat bone marrow aspirate and analysis by flow cytometry was performed. The plasma cell population was undetected at this time point. Arrows indicate the plasma cell population.
