# Supplementary material for: Treatment of Systemic Lupus Erythematosus using BCMA-CD19 Compound CAR
Source: Stem Cell Rev Rep. 2021 Aug 30;17(6):2120–3. doi: 10.1007/s12015-021-10251-6 (PMC8599262; doi:10.1007/s12015-021-10251-6)
Supplement: Supplementary file 2 — Supplementary file2 (DOCX 768 KB) [file 12015_2021_10251_MOESM2_ESM.docx]

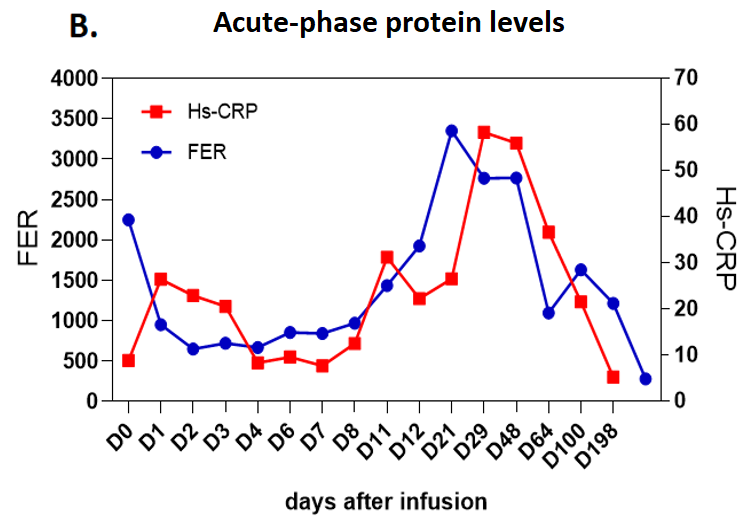

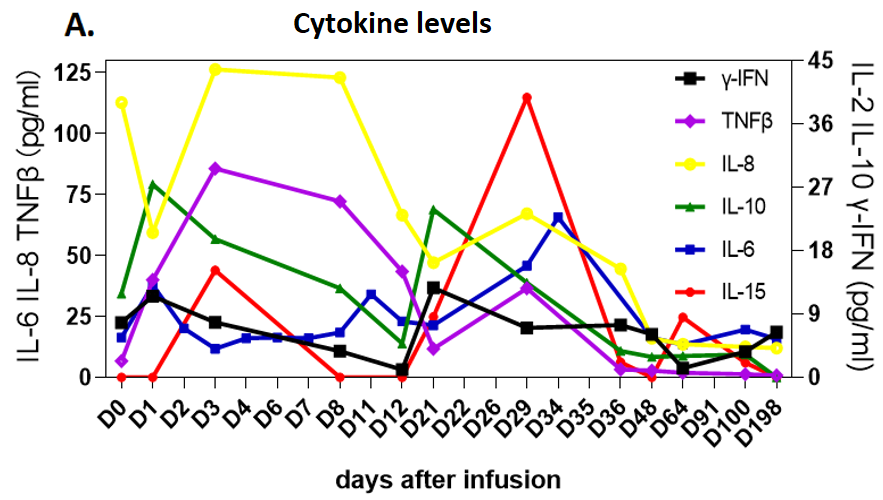


**Supplementary Figure 2**- **Levels of cytokines and acute-phase proteins following cCAR infusion**. (**A.**) Interferon-γ, TNF-β, IL-8, IL-10, IL-6, and IL-15 levels were followed over the course of cCAR treatment. (**B.**) The levels of two positive acute-phase proteins, high sensitivity C-reactive protein (Hs-CRP) and ferritin, were also measured after cCAR treatment.
